# Estimated hemodynamic response function parameters obtained from resting state BOLD fMRI signals in subjects with autism spectrum disorder and matched healthy subjects

**DOI:** 10.1016/j.dib.2018.04.126

**Published:** 2018-05-05

**Authors:** Wenjing Yan, D. Rangaprakash, Gopikrishna Deshpande

**Affiliations:** a AU MRI Research Center, Department of Electrical and Computer Engineering, Auburn University, Auburn, AL, USA; b Department of Psychiatry and Biobehavioral Sciences, University of California Los Angeles, Los Angeles, CA, USA; c Department of Psychology, Auburn University, Auburn, AL, USA; d Alabama Advanced Imaging Consortium, Auburn University and University of Alabama Birmingham, AL, USA; e Center for Health Ecology and Equity Research, Auburn University, AL, USA

## Abstract

In Functional magnetic resonance imaging (fMRI), the blood oxygen level dependent (BOLD) signal is modeled as a convolution of the hemodynamic response function (HRF) and the unmeasured latent neural signal. Although most cortical and subcortical brain regions share the canonical shape of the HRF, the temporal structure of HRFs are variable across brain regions and subjects. This variability is induced by both neural and non-neural factors. The variability between subjects can be examined by three parameters that characterize the HRF: response height (RH), time-to-peak (TTP) and full-width at half-max (FWHM). This data provides three HRF parameters at every voxel, obtained from Autism Spectrum Disorder (ASD) patients (*N* = 531), and matched healthy controls (*N* = 571). Since ongoing studies suggest that non-standard populations have important differences in their HRFs when compared with healthy control, this data set is valuable in studying variability of HRF in ASD group and inferring the underlying pathology that also affects the HRF. It also has implications for fMRI analyses like resting-sate connectivity analysis.

## Specifications Table

**Table:** 

Subject area	*Brain imaging*
More specific subject area	*Functional magnetic resonance imaging, hemodynamic variability, hemodynamic response function parameters, autism spectrum disorder*
Type of data	*Image: Three voxel-wise HRF parameters over the whole brain for every subject*
How data was acquired	*Specific MRI Scanners used by the 17 institutions that contributed the fMRI data*
Data format	*MAT-file (.mat)*
Experimental factors	*Two populations were considered: ASD patient and healthy controls*
Experimental features	*The parameters of HRF were estimated from resting-state fMRI data. The participants do not perform any specific task.*
Data source location	*Auburn, AL, United States of America (GPS coordinates: 32.586, − 85.494)*
Data accessibility	*Data is available at*https://drive.google.com/drive/folders/17Kd21LMaKO7eMyaihptyZx9zbeMXK2RG?usp=sharing

## Value of the data

• This data provides information of HRFs in autism spectrum disorder (ASD) patients and matched healthy controls. It could be used to assess the HRF variability in these two populations. Moreover, the shape of the HRF is affected by both neural and non-neural factors [1], [2], [3], [4], [5]. The data could be used in exploring the mechanism of the hemodynamic response through the HRF.
• In pathological populations, the non-neural factors that control the HRF shape also contribute to the brain pathology. For example, imbalances in GABA, nitric oxide, glutamate or serotonin in the ASD population may also impact the shape of the HRF [6]. Therefore, the data could be used in characterizing pathology in ASD.
• As mentioned above, the HRF shape could be related to ASD pathology. Therefore, the differences in HRF parameters between ASD patients and matched controls could be used in classifying these two populations.
• This data could be used to study the impact of HRF variability on resting-state fMRI data analyses such as effective and functional connectivity modeling [7].

## 1 Data

The data presents three HRF parameters (RH, TTP, and FWHM) at each brain voxel for 1102 subjects, which includes 531 ASD patients and 571 healthy controls. For each parameter in each subject, the 3-dimensional data consisted a volume dimension of 91 × 109 × 91. The 3D matrix was coregistered to the Montreal Neurological Institute (MNI) space with the voxel dimension of 2 × 2 × 2 mm^3^. The provided data is organized as six 4-dimensional matrices stored as two MAT-files (.mat). The parameters for the ASD and control groups were constructed as separate MAT-files. Each mat-file contain three 4D matrices, representing three HRF parameters (RH, TTP and FWHM) for every subject. The first dimension of the file represents the number of subjects and the remaining three dimensions represent the three spatial coordinates of each brain volume. Since the fMRI data used to estimate HRF parameters were provided by different institutions, a one-way ANOVA analysis was performed to investigate inter-site differences.

## 2 Experimental design, materials and methods

### 2.1 Participants

The resting-state fMRI data was obtained from the Autism Brain Imaging Data Exchange (ABIDE-1) database [8]. Seventeen different institutions contributed to this data set, including California Institute of Technology, Kennedy Krieger Institute, University of Leuven, Ludwig Maximilians University Munich, Oregon Health and Science University, University of Pittsburgh School of Medicine, Social Brain Lab UMC Groningen NIN, San Diego State University, Stanford University, Trinity College Dublin, University of California Los Angeles, University of Michigan, NYU Langone Medical Center, Olin, Institute of Living at Hartford Hospital, University of Utah School of Medicine, Yale Child Study Center, and Carnegie Mellon University. The resting-state fMRI data were acquired from 1102 subjects, including 531 individuals with ASD and 571 age- and sex-matched typical controls. Of these subjects, 739 were males and 363 were females. Local Institutional Review Boards (IRBs) approved the study protocol at each institution, the subjects provided informed consent and the data was fully anonymized in accordance with Health Insurance Portability and Accountability Act (HIPAA) guidelines. Details of acquisition, informed consent, and site-specific protocols are available at http://fcon_1000.projects.nitrc.org/indi/abide/.

### 2.2 FMRI data pre-processing

The pre-processing pipeline for this dataset was confined to the steps performed by ABIDE prior to publication of this public dataset. For each individual participant, the first four temporal image volumes were discarded. The remaining volumes were pre-processed with the following steps. Slice timing correction was performed first. For each volume, the signal measured in each slice was shifted relative to the slice that was acquired at the mid-point of each TR. All the images were realigned by using the six rigid body motion parameters. Spatial normalization was then performed and all the images were normalized to the Montreal Neurological Institute (MNI) template. In order to reduce the effects of head motion, motion regression was carried out by applying a 24-parameter (6 head motion parameters, 6 head motion parameters one time point before, and the 12 corresponding squared items) model [9]. Finally, white matter and cerebrospinal fluid signal regression were performed to reduce respiratory and cardiac confounds. All the procedures were performed on the Matlab^®^ platform using the Data Processing Assistant for Resting-State fMRI (DPARSF) toolbox [10], which is based on Statistical Parametric Mapping (SPM8) [11] and Resting-State fMRI Data Analysis Toolkit [12].

### 2.3 Acquiring the HRF parameters

We further studied a recent blind deconvolution method [13] to estimate HRF parameters from the pre-processed fMRI time series. The method assumes that the resting-state fMRI signal is driven by spontaneous neural events occurring at random times. It constructs pseudo neural events by identifying points of relatively large amplitude in the time series [14]. Then the pseudo-event onsets would be shifted with different delays to determine the onset that optimally fits the measured fMRI time series. Once the onsets were determined, voxel-specific HRFs were reconstructed from the raw BOLD signal by using Weiner deconvolution [15]. The three voxel-specific parameters that represented the shape of the HRF (RH, TTP, and FWHM) were obtained, and the latent neuronal events were estimated as well. This technique has been increasingly applied in fMRI studies, and the robust and valid estimation of HRF has been demonstrated [16], [17], [18], [22], [23].

Based on the HRF parameters obtained from the ASD and control populations, we further studied the systemic aberration of the HRF between these two populations, and the possible confounds introduced by the variable HRF in resting-state connectivity analysis. These findings have been published as a full article at NeuroImage: Clinical [19]. Recently, we have reported that HRF parameters in other disorders such as post-traumatic stress disorder are altered and they do confound functional connectivity estimates [20], [21], [24], [25]. Therefore, we believe that a similar investigation in ASD is warranted.
